# Exploring the association between internet addiction and time management among undergraduate nursing students

**DOI:** 10.1186/s12912-024-02273-5

**Published:** 2024-09-11

**Authors:** Heba Fakieh Mansy Ali, Marwa Abd-El-Gawad Mousa, Mohamed Hussein Ramadan Atta, Shadia Ramadan Morsy

**Affiliations:** 1 Nursing Education Department, Faculty of Nursing, Alexandria University, Alexandria City, Egypt; 2 Psychiatric and Mental Health Nursing Department, Faculty of Nursing, Alexandria University, Alexandria City, Egypt

**Keywords:** Internet addiction, Time Management, Undergraduate nursing students

## Abstract

**Background:**

Modern undergraduate nursing students face unique challenges as digital natives balancing internet activities with the substantial academic demands of nursing studies. Given the detrimental effects of internet addiction on students’ academic performance and well-being, having time management skills is crucial.

**Aims:**

To assess the prevalence and levels of internet addiction and time management and their association among undergraduate nursing students.

**Design:**

A cross-sectional, survey-based research design was used.

**Setting:**

The Faculty of Nursing at Alexandria University in Egypt.

**Subjects:**

A stratified random sample consisting of 825 undergraduate nursing students.

**Tools:**

The internet addiction test and time management questionnaire were utilized to collect data.

**Findings:**

Internet addiction was prevalent among 98.8% of students, with 56.0% exhibiting mild levels, 40.0% showing moderate levels, and 2.8% having severe levels. A statistically significant negative correlation was found between students’ internet addiction and overall time management (*r*= − 0.387, *p* < 0.001).

**Conclusion:**

A considerable level of internet addiction was revealed among the great majority of undergraduate nursing students; however, many students also demonstrated strong time management skills. Furthermore, internet addiction and overall time management were negatively associated, indicating that students with higher levels of internet addiction tend to have poorer time management abilities.

**Recommendations:**

Individual counseling and educational training programs should be developed to teach nursing students how to manage time and effectively plan internet usage.

**Supplementary Information:**

The online version contains supplementary material available at 10.1186/s12912-024-02273-5.

## Introduction

In the contemporary era marked by rapid technological advancements, the pervasive use of the internet is a reality that extends across various age groups. There are 5.3 billion regular users worldwide, amounting to 67.9% of the global population, of whom 22.8% are aged between 18 and 24 years old [1]. According to a report on internet usage in 2023 by the International Telecommunication Union, 79% of individuals between the ages of 15 and 24 regularly use the internet in Arab countries, 12 points higher than the average for the general population (65%). The report also mentioned that the overall rate of the Arab population using the internet in 2023 was 69%, with larger percentages among younger age cohorts, especially those aged between 15 and 24 years [2]. Specifically, at the beginning of 2023, Egypt had 80.75 million internet users [3]. According to the Central Agency for Public Mobilization and Statistics, youths between 18 and 29 comprised 21% of the Egyptian population, and 89% of these Egyptians regularly use the internet [4].

The internet provides resources for learning and connecting with others. However, while balanced use is generally considered to be harmless, excessive or problematic use can lead to many challenges, ranging from sleep disruptions and depleted energy to academic decline, social withdrawal, functional impairment, and even reduced life satisfaction [5, 6]. These adverse consequences pose a significant threat to the well-being of those affected, particularly young individuals and students who are vulnerable to developing internet addiction (IA), a growing psychiatric concern with detrimental impacts on various life aspects [7–9].

Internet addiction, also referred to as “problematic” or “compulsive” internet use, refers to a behavioral disorder characterized by excessive and uncontrollable preoccupations regarding internet usage and access that disrupts daily functioning and leads to negative consequences. These consequences affect individuals’ physical and mental health, relationships, and productivity [10, 11]. According to the *Diagnostic and Statistical Manual of Mental Disorders*, *Fifth Edition* (DSM-5), internet addiction is recognized as a potential mental health issue that can negatively impact individuals’ academic, social, and occupational functioning. The DSM-5 criteria offer a framework for understanding and diagnosing internet addiction, with a focus on criteria such as loss of control, continued overuse, and mood regulation [12]. However, Bottel et al. (2023) found that the criterion “jeopardizing” was the most significant predictor for internet addiction, followed by “loss of interest” and “continued overuse” [13].

It has been estimated that up to 7.02% of all people worldwide exhibit symptoms of internet addiction, with higher rates among younger age groups [14]. This phenomenon is widespread among students, who rely primarily on digital tools for academic and recreational purposes. The prevalence of internet addiction among student populations in developing countries, including nursing students, has been extensively documented in the literature [8, 15–22]. In Egypt, Rabea et al. (2023) reported that 61.2% of nursing students have severe internet addiction, and 21.5% have moderate levels [23].

The consequences of internet addiction extend beyond mere online activities, infiltrating students’ academic performance, mental health, and general health profiles [7, 9, 24, 25]. Research studies have shown that students categorized as being at risk of internet addiction exhibit poorer communication skills, experience more loneliness and isolation, and have higher levels of mental distress, anxiety, depression, interpersonal sensitivity, self-distraction, academic burnout, and difficulties regulating their emotions [8, 20, 26–31]. Such negative impacts are particularly concerning in the high-pressure context of nursing education, where academic achievement, psychological well-being, and coping strategies are crucial for success [23, 25, 32, 33].

Additionally, common physical problems of internet addiction include musculoskeletal issues, fatigue, visual discomfort, and even obesity [29]. Researchers have also suggested that internet addiction can significantly affect time management skills, leading to procrastination, decreased academic performance, and increased stress [29, 34]. Britton and Tesser (1991) stated that effective time management requires three essential skills: (1) *short-range planning skills* relate to organizing tasks for the short term, making to-do lists, setting priorities, and breaking down tasks into manageable steps; (2) *time attitude skills* relate to time consciousness, procrastination, and overall satisfaction with time use; and (3) *long-range planning skills* involve setting goals for the longer term, developing schedules, and tracking progress [35]. By adopting these time management skills, students can achieve greater efficiency in their academic performance [36].

Unfortunately, nursing students in today’s digital world face unique challenges in using their time efficiently, making accurate time management essential for academic achievement and career development. The easy accessibility of the internet can lead students to spend excessive time on online activities, negatively affecting their time management and consequently hindering their academic performance and well-being [33, 34, 36, 37]. According to Kutty et al. (2022), nearly half of university students reported spending more than eight hours on the internet daily for various purposes [38]. In addition, Han et al. (2023) estimated that a quarter of students use digital devices for 7–9 h each day [39]. A study conducted by Soares et al. (2023) further corroborated these concerns, whereby university students reported that managing their time is a significant challenge due to distractions caused by digital activities. Specifically, the primary sources of distraction were related to cell phones, social media, websites, and other internet -related activities [40].

Given the potential harm of internet addiction, particularly for students, time management skills are crucial. Effective time management allows students to prioritize tasks, manage online activities, and optimize a healthy work-life balance. This is especially vital for nursing students, who face a demanding workload of classes, assignments, clinical rotations, and extracurricular activities [32, 41]. Studies have shown a clear correlation between solid time management skills and better academic performance, stress reduction, improved psychological well-being and mental health, and increased preparedness of future nurses for their professional roles requiring deadlines and multiple tasks [42, 43].

This study’s theoretical framework is grounded in the Theory of Planned Behavior (TPB) [44], which posits that attitudes, subjective norms (e.g., perceived social pressure), and perceived behavioral control are key determinants of human behavior. This framework is particularly relevant in explaining how undergraduate nursing students’ beliefs, social influences, and perceived control over their internet use and time management practices shape their behaviors [45, 46]. Furthermore, the TPB’s focus on cognitive, social, and self-regulatory factors aligns well with the potential influences on nursing students’ behaviors. This alignment strengthens the study’s ability to identify effective interventions that promote healthier internet habits and improved time management skills [47].

### Significance of the study

Although prior research has investigated students’ internet addiction and its relationship with various factors, examining internet addiction’s relationship with time management remains under-researched, particularly among Egyptian undergraduate nursing students. Existing research highlighted the negative association between internet addiction and students’ outcomes in clinical self-efficacy, academic experience, academic performance, and communication skills [48]. While other studies suggested a negative correlation between internet addiction and time management in nursing students [29], a broader understanding of this link is needed. The current study seeks to address this gap in existing knowledge regarding internet addiction and time management in Egyptian nursing students.

Investigating the intricate association between these two variables is crucial, as integrating digital technology in healthcare has made practical time management skills essential for nurses. Managing electronic health records, accessing evidence-based resources, and communicating efficiently with healthcare teams requires strong time management and digital literacy skills. Addressing the relationship between internet addiction and time management is crucial to prepare future nurses for integrating technology into their practice while maintaining balanced lives. This understanding can improve the quality of nursing education, the healthcare profession, and overall patient care and outcomes.

By bridging this knowledge gap, the present study aims to provide valuable insights into this crucial aspect of nursing education and contribute to the broader understanding of digital challenges among Egyptian undergraduate nursing students. Ultimately, this study’s outcomes could lead to improved academic performance, reduced stress levels, and better preparation of future nurses for the profession’s demands.

## Aims

The aims of this study were to:

- Assess the prevalence and levels of internet addiction and levels of time management.
- Explore the negative association between internet addiction and time management among undergraduate nursing students.

## Methods

### Design

The cross-sectional survey-based research design used in the current study adhered to the Strengthening the Reporting of Observational Studies in Epidemiology (STROBE) [Supplementary 1].

### Setting

The study was conducted at Alexandria University’s Faculty of Nursing, which is affiliated with the Ministry of Higher Education in Egypt. The Faculty of Nursing employs a credit-hour system for undergraduate and graduate programs. To obtain a baccalaureate degree in nursing sciences, an undergraduate student has to finish four academic levels and a comprehensive curriculum extended over eight semesters.

### Participants and recruitment

A stratified random sample was employed to recruit a representative sample of 825 undergraduate nursing students from a population of 3539 enrolled across four academic levels (first, third, fifth, and seventh semesters) during the first academic term of the 2022–2023 academic year. The Epi Info 7 software program determined the stratified random sample size. The parameters were as follows: the population size is 3539 undergraduate students; the expected frequency is 50%; the margin of error is 5%; and the confidence coefficient is 95%. The program estimated a sample size of 825 nursing students, allocated proportionally across the four academic semesters, as presented in Table 1.

**Table 1:** Sample allocation of nursing students using a stratified random sampling technique

Academic semester	Actual number of students	Recruited number of students
First	1531	337
Third	890	219
Fifth	626	203
Seventh	492	66
***Total***	***3539***	***825***

The stratified random sample selection process utilized a blind approach, incorporating a systematic randomized technique to identify participants from the overall pool of students. First, a comprehensive list containing all students’ names from each stratum and relevant data was compiled and digitized into a computer-generated randomization list program. This randomization program recruited selected students from this list through random sampling to ensure fairness and minimize bias. Each randomly recruited student was screened to identify those meeting the predetermined inclusion criteria, who were then contacted to assess their willingness to participate.

### Measures

The original English language tools described below were administered to study participants. Their academic program is English-medium, and all of them speak English fluently, as per the academic admission requirements to study health sciences.

#### Internet Addiction Test (IAT)

The IAT was designed by Young (1998) to assess the prevalence rate and levels of internet addiction. The test contains 20 self-rated items, each of which is scored on a six-point scale ranging from 0 (“not applicable”) to 5 (“always”) [49]. The total score runs from 0 to 100 points, with higher scores reflecting greater severity of internet addiction. The levels of addiction are categorized as follows: scores ranging from 0 to 30 points indicate an average level of internet usage (absence of addiction), scores of 31 to 49 indicate mild addiction, scores of 50 to 79 indicate moderate addiction, and scores of 80 to 100 indicate severe addiction [50]. The IAT was validated for nursing students in Egyptian studies. The internal consistency of the test was measured using Cronbach’s alpha method, resulting in a coefficient value of 0.95 and 0.93, according to Ibrahim (2015) and Younis et al. (2020), respectively [51, 52]. In the current study, Cronbach’s alpha coefficient of the IAT is 0.83 (Table 2).

**Table 2:** Reliability statistics of the present study instruments

Instruments	No. of Items	Cronbach’s alpha coefficient
**Internet Addiction Test (IAT)**	**20**	**0.83**
**Time Management Questionnaire (TMQ):**	**18**	**0.88**
Short-Range Planning Subscale	7	0.89
Time Attitudes Subscale	6	0.91
Long-Range Planning Subscale	5	0.77

#### Time Management Questionnaire (TMQ)

The TMQ was constructed by Britton and Tesser (1991) to assess the time management skills of university students. Comprising 18 questions, the TMQ is subdivided into three subscales: *Short-Range Planning* (7 questions), *Time Attitudes* (6 questions), and *Long-Range Planning* (5 questions). A five-point Likert scale was used in the TMQ, varying from 1 (“never”) to 5 (“always”) [35]. The total score ranges from 18 to 90. Total scores from 0 to 54, 55 to 69, and 70 to 90 indicate low, moderate, and high time management skills (respectively). The subscale scores range between 7 and 35 for the Short-Range Planning Subscale, 6 and 30 for the Time Attitudes Subscale, and 5 and 25 for the Long-Range Planning Subscale. Higher total and subscales scores indicate better time management skills [53]. The TMQ exhibits internal consistency, as evidenced by Cronbach’s alpha coefficient values. Specifically, the entire inventory demonstrates a coefficient of 0.77, while the Short-Range Planning Subscale displays a coefficient of 0.81. The Time Attitudes Subscale and Long-Range Planning Subscale manifest coefficients of 0.57 and 0.48, respectively [54]. The TMQ proved to be reliable in the present study, as indicated by the reliability statistics illustrated in Table 2.

In addition, a nursing student’s personal and academic data sheet was attached to the previously mentioned instruments. It included items related to the student’s sex, age, current academic semester, number of registered hours for the current semester, and last obtained Grade Point Average (GPA) [Supplementary 2].

### Ethical considerations

Before conducting the research, approval was obtained from the Research Ethics Committee (REC) of the Faculty of Nursing at Alexandria University (IRB00013620; Serial Number: 2022-9-75). The Dean of the Faculty of Nursing at Alexandria University granted official permission. As per the ethical requirements, each potential participant was informed in full about the nature of the study, including its aims, the voluntary nature of their participation, and the right to withdraw without penalty. They were informed that all data would be anonymized and assured complete confidentiality and anonymity.

### Data collection

Five experts in nursing education and psychiatric and mental health nursing were consulted to assess the content validity of the IAT and TMQ. Based on the experts’ assessment, the instruments demonstrated validity. A pilot study was conducted on 10% of undergraduate nursing students to evaluate the instruments’ feasibility, clarity, and applicability. Notably, these students were excluded from the study subjects. The reliability of both instruments was also assessed using Cronbach’s alpha method, and the reliability statistics are presented in Table 2.

Before collecting the data, the researchers conducted group interviews with the recruited students in their classrooms, either at the end of their lectures or clinical days. The researchers introduced themselves to the students, explained the study’s purpose, and ensured that ethical approval was affirmed. The questionnaires were distributed to collect students’ personal and academic information and assess their internet addiction and time management levels. The researchers were present during the data collection to provide additional clarification.

### Data analysis

The collected data were entered into the computer for analysis using IBM SPSS Software Package Version 20.0. (Armonk, NY: IBM Corp). Cronbach’s alpha coefficient was employed to assess reliability statistics. Descriptive statistics were employed for qualitative data in numbers and percentages. The Kolmogorov-Smirnov test was utilized to verify the normality of distribution. Quantitative data were described using range (minimum and maximum), mean, standard deviation (SD), and median values.

The F-test for analysis of variance (ANOVA) was used to compare more than two independent means. The Pearson correlation coefficient was used to examine the correlation between normally distributed quantitative variables. The significance of the obtained results was determined at a *p*-value ≤ 0.001.

## Results

Table 3 presents the personal and academic characteristics of undergraduate nursing students. It can be seen that 69.9% of students were females, and more than half of the sample were under 20 years old (56.8%); students’ ages ranged between 18 and 24 years, with a mean age of 19.40 ± 1.76 years. In terms of their current semester since enrollment, the largest cohort (40.8%) were in their first semester, commensurate with their age, followed by about a quarter each in their third (26.5%) and fifth (24.6%) semesters, and a smaller proportion (8.0%) in their seventh semester. The vast majority of students were registered for 16 to 18 h for the current semester (83.9%). Regarding the students’ last obtained GPA, 45.7% of students obtained B- to B+, 36.3% obtained A- – A, and 16.8% obtained C- – C+.

**Table 3:** Distribution of the undergraduate nursing students according to their personal and academic characteristics

Personal and academic characteristics	Nursing students (*n* = 825)	
	No.	%
**Sex**		
Female	577	69.9
Male	248	30.1
**Age (in years)**		
< 20	469	56.8
≥ 20	356	43.2
*Min. – Max.*	*18.0–24.0*	
*Mean ± SD*	*19.40 ± 1.76*	
*Median*	*19.0*	
**Academic semester**		
First	337	40.8
Third	219	26.5
Fifth	203	24.6
Seventh	66	8.0
**Number of registered hours for the current semester**		
< 12 h	4	0.5
12–15	71	8.6
16–18	692	83.9
19–21	58	7.0
**Last obtained GPA** ^*^	(*n* = 488)	
A- – A	177	36.3
B- – B+	223	45.7
C- – C+	82	16.8
D- D+	4	0.8
F	2	0.4

SD: Standard deviation

^*^Last obtained GPA for all students except those currently in their first semester

Table 4 shows the undergraduate students’ levels of internet addiction as measured by the IAT. Internet addiction was prevalent among 98.8% of students, of whom 56.0% exhibited a mild level, 40.0% showed a moderate level, and 2.8% had severe internet addiction. Only 1.2% of the students were found to have an average level of internet usage. The total students’ internet addiction score ranged between 20.0 and 92.0, with a mean score of 46.68 ± 11.68 and a median of 44.0. Additionally, the students recorded a mean percent score of 72.36 ± 17.97.

**Table 4:** Distribution of the undergraduate nursing students according to their levels and scores of internet addiction (*n* = 825)

Levels of internet addiction	No.	%
Normal / absence of addiction (0–30)	10	1.2
Mild (31–49)	462	56.0
Moderate (50–79)	330	40.0
Severe (80–100)	23	2.8
***Total score (0–100)***		
*Min. – Max.*	*20.0–92.0*	
*Mean ± SD* *Median*	*46.68 ± 11.68* *44.0*	
***% Score***		
*Min. – Max.*	*10.0–90.0*	
*Mean ± SD* *Median*	*33.35 ± 14.60* *30.0*	

SD: Standard deviation

Table 5 shows the undergraduate students’ time management levels (as measured by the TMQ). 62.4% of students had a high level of time management, followed by a moderated level (35.8%). Only 1.8% of students had a low level of time management. Students’ time management scores ranged between 18.0 and 90.0, with a mean score of 74.34 ± 9.51, a median of 75.0, and a mean percent score of 69.69 ± 15.99.

**Table 5:** Distribution of the undergraduate nursing students according to their levels and scores of time management (*n* = 825)

Levels of time management	No.	%
Low (0–54)	15	1.8
Moderate (55–69)	295	35.8
High (70–90)	515	62.4
***Total score (18–90)***		
*Min. – Max.*	*18.0–90.0*	
*Mean ± SD* *Median*	*74.34 ± 9.51* *75.0*	
***% Score***		
*Min. – Max.*	*0.83–100.0*	
*Mean ± SD* *Median*	*69.69 ± 15.99* *71.0*	

SD: Standard deviation

Table 6 illustrates the students’ scores on the three subscales of time management skills. The scores for the Short-Range Planning subscale ranged from 7.0 to 35.0, with a mean of 29.52 ± 4.70 and a median of 30.0. On the Time Attitudes subscale, scores ranged from 6.0 to 30.0, with a mean of 20.65 ± 2.68 and a median of 21.0. Finally, the students had scores ranging from 5.0 to 25.0 on the Long-Range Planning subscale, with a mean of 13.93 ± 1.32 and a median of 21.0.

**Table 6:** Undergraduate nursing students’ scores on the subscales of time management skills (*n* = 825)

Time management skills	Min. – Max.	Mean ± SD	Median
Short-Range Planning Subscale (7–35)	7.0–35.0	29.52 ± 4.70	30.0
Time Attitudes Subscale (6–30)	6.0–30.0	20.65 ± 2.68	21.0
Long-Range Planning Subscale (5–25)	5.0–25.0	13.93 ± 1.32	14.0
***Total score (18–90)***	***18.0–90.0***	***74.34 ± 9.51***	***75.0***

SD: Standard deviation

Table 7 shows the relationship between internet addiction levels and undergraduate nursing students’ time management skills. A statistically significant relationship between the levels of internet addiction and overall time management was evident (F = 43.247, *p* < 0.001). Overall time management scores decline with increasing internet addiction. Students with normal internet use have a higher mean score (82.73 ± 5.86) compared to those with mild (76.78 ± 8.16) or moderate (70.18 ± 10.7) levels of internet addiction. In contrast, those with severe internet addiction have the lowest mean score (65.09 ± 5.66).

**Table 7:** Relationship between levels of internet addiction and time management skills of the undergraduate nursing students (*n* = 825)

Time management skills	Levels of internet addiction				F	*p*
	Normal (*n* = 10) Mean ± SD	Mild (*n* = 462) Mean ± SD	Moderate (*n* = 330) Mean ± SD	Severe (*n* = 23) Mean ± SD		
Short-Range Planning (Range = 7–35)	30.64 ± 4.02	27.63 ± 4.96	25.21 ± 3.42	19.34 ± 5.84	42.342^*^	< 0.001
Time Attitudes (Range = 6–30)	26.95 ± 2.85	21.66 ± 2.48	18.93 ± 3.02	16.68 ± 4.18	95.087^*^	< 0.001
Long-Range Planning (Range = 5–25)	21.32 ± 3.77	20.58 ± 2.49	17.28 ± 1.41	15.11 ± 1.16	87.941^*^	< 0.001
*Overall time management (Range = 18–90)*	*82.73 ± 5.86*	*76.78 ± 8.16*	*70.18 ± 10.7*	*65.09 ± 5.66*	*43.247* ^*^	*< 0.001*

SD: Standard deviation

F: F test (ANOVA)

P: *p*-value for correlation between students’ IA and TM skills

* Statistically significant at *p* ≤ 0.001

The same was also true with levels of internet addiction and all time management skills (*p* < 0.001 each). As the level of internet addiction increases (from normal to severe), the scores for all time management skills (short-range planning, time attitudes, long-range planning, and overall time management) consistently decrease. Specifically, students with normal internet use have a higher mean score on the short-range planning skill (30.64 ± 4.02) compared to those with mild (27.63 ± 4.96) or moderate (25.21 ± 3.42) levels of internet addiction. In contrast, those with severe internet addiction have the lowest (19.34 ± 5.84).

A similar pattern is observed for time attitudes, where students with normal internet use also score higher (26.95 ± 2.85) in time attitudes compared to other groups (21.66 ± 2.48 for mild, 18.93 ± 3.02 for moderate, and 16.68 ± 4.18 for severe). The mean scores for long-range planning skills also decrease with increasing internet addiction, where students with normal internet use have the highest mean score (21.32 ± 3.77). In contrast, severely addicted students have the lowest mean score (15.11 ± 1.16).

Table 8 presents the correlation matrix between the mean scores of students’ internet addiction and their time management skills. A statistically significant negative correlation was found between students’ internet addiction and overall time management (*r* = − 0.387, *p* < 0.001). Additionally, internet addiction is negatively and significantly correlated with the three skills of time management (*p* < 0.001 for each skill).

**Table 8:** Correlation matrix between mean scores of the undergraduate nursing students’ internet addiction and their time management skills (*n* = 825)

Time management skills	Internet addiction
Short-Range Planning	*r* = − 0.377* *p* < 0.001
Time Attitudes	*r* = − 0.390* *p* < 0.001
Long-Range Planning	*r* = − 0.276* *p* < 0.001
***Overall time management***	r = − 0.387* ***p < 0.001***

R: Pearson correlation coefficient

* Statistically significant at *p* ≤ 0.001

## Discussion

The ubiquitous use of the internet, especially social media sites accessed via smartphones, has pervaded young people’s lives, and this can be compounded in the academic context by the increasing popularity of online education, especially since COVID-19 lockdowns. Such factors have led to a rise in internet addiction, which can negatively impact students’ well-being. However, proper time management can help students allocate their study goals better, mitigating the negative impacts of internet addiction and improving their quality of life and academic achievement [36, 42, 43, 55, 56]. This study focuses on determining the challenges experienced by a sample of Egyptian undergraduate nursing students, particularly regarding internet addiction and time management. It provides insight into the prevalence and severity of internet addiction among these students.

The results revealed that most undergraduate nursing students exhibited symptoms of internet addiction, generally ranging from mild to moderate but extending to severe in some cases. These findings are consistent with the literature on nursing students in developing countries, including Egypt [8, 15, 17–22, 57, 58]. Hassan et al. (2020) reported different results and found that only around one-quarter of participants displayed symptoms of internet addiction. The authors attributed the differences in their results from other studies to various factors, including cultural differences, variations in diagnostic criteria and assessment questionnaires used for diagnosis, and highly selective samples in online surveys [16].

Nevertheless, the prevalence of moderate and severe levels of internet addiction in the current study is relatively low compared to previous studies. Specifically, among the students with internet addiction, more than half showed mild internet addiction, while just over a third of them exhibited a moderate level of internet addiction, and only a small minority of students displayed severe addiction. These results may reflect the particular awareness of *nursing* students (i.e., as aspiring healthcare professionals) of the harmful effects of internet misuse on physical, psychological, and social health. This knowledge may enable students to use the internet and social networking sites more responsibly and effectively than non-healthcare students and the general population. Younis et al. (2020) also found that most nursing students had mild and moderate levels of internet addiction, while a minority had severe internet addiction [52]. On the other hand, the results of the current study differ from the study of Kumar and Mondal (2018), who reported a higher prevalence of severe internet addiction among general (i.e., non-healthcare) students [28].

Lin et al. (2016) argued that internet addiction negatively impacts students’ academic performance, mental health, and social relationships [59]. Several educational factors contribute to this issue; academic pressure compels students to spend long hours online to keep up with their coursework, leading to excessive research and procrastination. In addition, poor time management skills can make the internet an attractive distraction from demanding schedules that include clinical rotations, lectures, and study sessions [60]. Social media platforms and entertainment websites often lead to prolonged internet use and addiction (and indeed are designed for this commercial purpose). Moreover, lacking social support pushes students to seek connections through online communities and social media platforms, which can result in addictive behaviors and negatively impact face-to-face interactions. Lastly, technological advancements, such as smartphones and laptops and increasingly ubiquitous mobile internet access, provide constant internet access, making it difficult for individuals to disconnect and contributing to problematic internet use through increased multitasking behaviors [59, 60].

Research evidence highlights the importance of time management skills for nursing students in this digital era. These skills are crucial for academic achievement, stress reduction, improved psychological well-being, and career development [36, 42, 55]. Time management skills are also critical for students during their clinical rotations, specifically shaping their learning experiences, stress levels, and overall performance [61, 62]. Effective time management allows nursing students to balance clinical responsibilities, academic demands, and personal lives efficiently. Proper time management enables nursing students to engage in clinical rotations, allocating sufficient time for hands-on practice, observation, and interaction with patients and healthcare professionals [63].

By effectively managing their time, students can participate in various clinical tasks, procedures, and simulations, which are essential for developing clinical skills and critical thinking abilities. Additionally, good time management helps students stay organized and focused on their learning objectives, ensuring steady progress throughout their clinical rotations without falling behind. Time management is closely linked to academic success in education, as students who allocate dedicated study hours alongside clinical practice are more likely to grasp complex concepts, retain information effectively, and perform well in exams and assessments [64]. In the healthcare setting, time management directly impacts clinical outcomes. Efficient time management is essential for healthcare professionals to provide optimal patient care, minimize errors, and enhance clinical competencies [65].

The present study confirms that undergraduate nursing students at the Faculty of Nursing (Alexandria University) possess a high level of overall time management, including skills related to short-term planning, long-term planning, and time attitudes. These results may be attributed to the challenging and intensive nature of undergraduate nursing programs, known for their demanding coursework, clinical rotations, and exams. The rigorous nature of these programs requires students to manage their time effectively to meet deadlines, study adequately, and fulfill clinical responsibilities. Such academic pressure can inadvertently cultivate practical time management skills as students learn to optimize their limited time. This aligns with the results of previous studies, which reported that undergraduate students generally exhibit moderate to high levels of time management behavior [37, 41, 43, 56]. According to Khan et al. (2019), these high levels of time management may be due to semester-based scheduling, which allows students to plan and prioritize their tasks according to their importance and achieve short- and long-term goals [43]. However, time management skills among nursing students are not universal. Based on the findings of Siddiqi and Memon (2016), out of their studied sample, 15% of students experienced severe time management difficulties, while 82% faced moderate problems [33].

Furthermore, the present study reveals a significant negative correlation between overall internet addiction and overall time management among undergraduate nursing students. This suggests that as internet addiction levels increase, time management decreases, and vice versa. The study has also detected significant relationships between levels of internet addiction and time management skills. Students with lower internet addiction levels consistently scored higher in all time management skills, including short-range planning, time attitudes, and long-range planning. Therefore, it can be inferred that the less severe the levels of internet addiction are, the better the time management skills, reinforcing the strong association between the two variables. These results emphasize the potential impact of internet addiction on students’ ability to manage their time effectively. Previous research has also supported the interdependence of internet addiction and time management, indicating that changes in one can affect the other. It has been demonstrated that students who spend more time on the internet have difficulty managing time, and time spent on the internet is a significant indicator of internet addiction [33, 56, 66, 67].

However, it is essential to note that the high prevalence of mild levels of internet addiction among nursing students observed in the current study may not necessarily contradict the findings on high time management skills. The results suggest that students with high time management skills can effectively manage their internet usage, preventing it from significantly impacting their academic performance or well-being. This is supported by the high levels of time management observed among nursing students in the study. It appears that students can balance their online activities with other responsibilities, even when experiencing mild addiction. These findings suggest the existence of a complex interplay between internet addiction and time management in nursing students.

### Strengths and limitations of the study

This study benefits from several strengths. First, stratified randomization ensured a representative sample of the undergraduate nursing student population, enhancing the reliability and generalizability of the findings. Second, the survey-based approach facilitated large-scale data collection, reaching diverse students. This broadens the understanding of this population’s association between internet addiction and time management. Finally, the study sheds light on a contemporary issue by examining the impact of internet addiction on students’ time management skills. These valuable results contribute to the ongoing discussion on internet addiction and offer insights for developing potential interventions.

However, this study acknowledges some limitations. Firstly, the focus on undergraduate nursing students in an Egyptian context restricts the generalizability of the findings to other populations or academic disciplines. In particular, the nature of the academic specialty involved (i.e., nursing) indicates that participants are axiomatically more health-conscious, and general students may have exhibited less healthy behaviors and outcomes. Future research could explore the impact of cultural factors and broaden the participant pool. Secondly, using self-reported data introduces the potential for social desirability bias, whereby participants may underreport negative behaviors like internet addiction. Future studies could consider incorporating objective measures alongside self-reports. Finally, the cross-sectional design limits the ability to establish causal relationships between internet addiction and time management. Longitudinal studies would be beneficial to explore how internet addiction might influence time management skills over time.

## Conclusion

It can be concluded that internet addiction is prevalent among most undergraduate nursing students at the Faculty of Nursing at Alexandria University, with varying degrees of severity ranging from mild to moderate to severe. Among those who face the challenge of internet addiction, more than half showed mild internet addiction, and just over a third exhibited a moderate level of addiction. Only a small minority exhibited severe addiction. However, the students demonstrated high levels of time management and its three skills, including short-term and long-term planning and time attitudes. Furthermore, the student’s overall internet addiction was negatively correlated with their time management. All students’ levels of internet addiction were also significantly related to all time management skills, highlighting the potential impact of internet addiction on students’ ability to manage their time effectively.

### Implications

The current study results propose several recommendations for practice, education, policy, and research. In practice, it is crucial to implement routine screening for internet addiction among undergraduate nursing students to ensure early identification and timely intervention. Individual counseling and educational training programs can equip students with effective time management skills and internet usage.

In education, it is recommended that modules on internet addiction prevention and management be integrated into the nursing curriculum. These modules should address responsible internet use, the consequences of excessive consumption, and internet addiction management strategies. Additionally, training programs for faculty members can enhance their awareness of internet addiction and equip them with the skills to support their students in regulating internet use effectively.

Policy recommendations suggest that educational institutions consider developing and enforcing responsible internet use policies and support programs, such as workshops and seminars on safe internet usage and time management strategies.

Finally, future research is necessary to explore the broader impact of internet addiction on students. This includes investigating the relationship between internet addiction, time management, and other factors influencing academic performance and well-being. Studies on the effectiveness of various interventions for managing internet addiction and enhancing time management skills among nursing students are also essential. Comprehensive research is needed to understand the multifaceted consequences of internet addiction on mental health, academic success, and social interactions.

## Electronic supplementary material

Below is the link to the electronic supplementary material.

Supplementary Material 1

Supplementary Material 2

## Data Availability

The datasets used and/or analyzed during the current study are available from the corresponding author on reasonable request.
