# Supplementary material for: Exploring the association between internet addiction and time management among undergraduate nursing students
Source: BMC Nurs. 2024 Sep 11;23:632. doi: 10.1186/s12912-024-02273-5 (PMC11389558; doi:10.1186/s12912-024-02273-5)
Supplement: Supplementary file 2 — Supplementary Material 2 [file 12912_2024_2273_MOESM2_ESM.docx]

**Personal and academic data**

**I.Personal data**

1. Sex: Male ( ) Female ( )
2. Age: ………………………………..

**II.Academic data**

1. Academic Semester:
2. First
3. Third
4. Fifth
5. Seventh

4.Number of registered hours for the current semester: …………………………………………………………………

5. Last obtained GPA:

.…………………………………………………………….
